# A meta-analysis of randomized controlled trials on the use of statins in septic patients

**DOI:** 10.1186/cc13437

**Published:** 2014-03-17

**Authors:** G Landoni, L Nobile, D Febres, E Frati, N Villari, AL Di Prima, R Dossi, L Pasin

**Affiliations:** 1 Vita-Salute San Raffaele University, Milan, Italy

## Introduction

Beneficial effects of 3-hydroxy-3-methylglutaryl coenzyme A (HMG-CoA) reductase inhibitors (statins) on vascular diseases have been demonstrated in several clinical trials. Recently discovered anti-inflammatory effects of statins seem to have an important role in counteracting the harmful effects of sepsis on the coagulation system. Many epidemiologic studies evidence that statin treatment may be associated with a better prognosis in severe bacterial infections, and a recent randomized trial found a reduced mortality in the statin group. We decided to perform a meta-analysis of all randomized controlled trials ever performed on statin therapy in septic patients to evaluate their effect on survival.

## Methods

Four trained investigators searched and assessed pertinent studies in BioMed Central, PubMed, Embase and the Cochrane Central Register (divergences resolved by consensus). Inclusion criteria were: random allocation to treatment; comparison of statins versus any comparator in septic patients. Exclusion criteria were: duplicate publications; nonadult studies; no data on main outcomes.

## Results

Data from 650 patients in five randomized controlled studies were analyzed. Overall analysis showed no difference in mortality between patients receiving statins (44/322 (14%)) versus control (50/328 (15%)), RR = 0.90 (95% CI 0.65 to 1.26), *P *= 0.6.

## Conclusion

Scientific evidence for the role of statins in septic patients is still limited and larger randomized trials should be performed on this topic. Published data, summarized by this meta-analysis of randomized trials, show that statin therapy has no detrimental effect on survival in the overall population of adult septic patients.

